# Fabrication and Characterization of MoS_2_/h-BN and WS_2_/h-BN Heterostructures

**DOI:** 10.3390/mi11121114

**Published:** 2020-12-16

**Authors:** Tao Han, Hongxia Liu, Shupeng Chen, Yanning Chen, Shulong Wang, Zhandong Li

**Affiliations:** 1 Key Laboratory for Wide-Band Gap Semiconductor Materials and Devices of Education, School of Microelectronics, Xidian University, Xi’an 710071, China; 15639119745@163.com (T.H.); slwang@xidian.edu.cn (S.W.); dong2890530@163.com (Z.L.); 2 State Grid Key Laboratory of Power Industrial Chip Design and Analysis Technology, Beijing Smart-Chip Microelectronics Technology Co., Ltd., Beijing 100192, China; chenyanning@sgitg.sgcc.com.cn

**Keywords:** APCVD, MoS_2_/h-BN heterostructure, WS_2_/h-BN heterostructure, spectral characteristics

## Abstract

The general preparation method of large-area, continuous, uniform, and controllable vdW heterostructure materials is provided in this paper. To obtain the preparation of MoS_2_/h-BN and WS_2_/h-BN heterostructures, MoS_2_ and WS_2_ material are directly grown on the insulating h-BN substrate by atmospheric pressure chemical vapor deposition (APCVD) method, which does not require any intermediate transfer steps. The test characterization of MoS_2_/h-BN and WS_2_/h-BN vdW heterostructure materials can be accomplished by optical microscope, AFM, Raman and PL spectroscopy. The Raman peak signal of h-BN material is stronger when the h-BN film is thicker. Compared to the spectrum of MoS_2_ or WS_2_ material on SiO_2_/Si substrate, the Raman and PL spectrum peak positions of MoS_2_/h-BN heterostructure are blue-shifted, which is due to the presence of local strain, charged impurities and the vdW heterostructure interaction. Additionally, the PL spectrum of WS_2_ material shows the strong emission peak at 1.96 eV, while the full width half maximum (FWHM) is only 56 meV. The sharp emission peak indicates that WS_2_/h-BN heterostructure material has the high crystallinity and clean interface. In addition, the peak position and shape of IPM mode characteristic peak are not obvious, which can be explained by the Van der Waals interaction of WS_2_/h-BN heterostructure. From the above experimental results, the preparation method of heterostructure material is efficient and scalable, which can provide the important support for the subsequent application of TMDs/h-BN heterostructure in nanoelectronics and optoelectronics.

## 1. Introduction

The two-dimensional van der Waals (2D vdWs) heterostructure materials have attracted research interest from researchers, and the controlled stacking of different 2D materials would greatly expand the type and application of heterostructures, which is due to the unique planar structure, excellent electrical and optical properties [1,2]. As the representative transition metal dichalcogenides (TMDs) materials, MoS_2_ and WS_2_ materials show the direct optical band gap, which has the significant photoluminescence (PL) intensity [3]. The band gap and dielectric constant of hexagonal boron nitride (h-BN) material, respectively, are 6 eV and 4, which has the excellent physical characteristics and atomic surface flatness [4]. For the inherent properties exploration of atomic layer materials, h-BN material is used as the most suitable substrate, and the performance can be improved when TMDs materials are stacked on the insulating h-BN substrate. Besides, the clean and flat heterojunction interface with the low-density charged impurities and dangling bonds can be formed [5,6]. The TMDs/h-BN vertical heterostructure materials can provide the unique platform, which can explore the unique phenomena of condensed state physical and electrical properties [7,8]. Based on the vdW interlayer coupling interaction, various vdW heterostructures exhibit unique quantum phenomena, which can be widely used in field effect transistors [9], sensors [10,11] and photodetectors [12].

There are many unexplored problems in the interlayer coupling of TMDs/h-BN heterostructures, and it is necessary to have a better understanding and application of vdW heterostructure [13,14]. The heterostructure combination of any layered materials can be prepared by transfer method, so the devices with new functions and characteristics are also realized by combining the different characteristics materials [15,16]. For the mass production of heterostructure materials, the mechanical peeling stack method is not suitable, due to the lower yield [17]. During the transfer processes, it can introduce the impurity contamination at the interface between h-BN and Graphene, and it can be severely limited by the interfacial contamination, poor interlayer contact and insufficient production scale, which would decrease the interlayer interaction [18,19,20]. Additionally, the method has a great impact on the properties of heterostructures. It is a more scalable and controllable method when 2D layered materials directly grown on another layer of material, which can produce the clean interface [21,22]. During the preparation of vdW heterostructure, 2D h-BN material has the flat surface and charge uniformity, it can achieve a cleaner interface, which is a good ideal insulating substrate [23,24,25]. The large area, continuous, uniform, and controllable preparation of TMDs/h-BN heterostructure is still a huge challenge. Therefore, it is necessary to develop a general construction method, which can use the same preparation processes to arbitrarily stack high-quality layered materials.

The research mainly includes the following parts. First, the h-BN material on Pt substrate is prepared by CVD method, which is transferred to SiO_2_/Si substrate. To confirm the existence of h-BN material, the spectral characteristics of h-BN materials with different layers are then tested and analyzed, which can provide a good foundation for the preparation of heterostructures. Next, MoS_2_ and WS_2_ materials are directly grown on the insulating h-BN substrate by APCVD method, which can complete the preparation of MoS_2_/h-BN and WS_2_/h-BN hetero-structures. Subsequently, the test characterization of MoS_2_/h-BN and WS_2_/h-BN vdW heterostructure materials can be accomplished by optical microscope, AFM, Raman and PL spectroscopy. Finally, the above characteristics would facilitate the preparation of TMDs/h-BN vdW heterostructure, which would also promote the application of TMDs/h-BN heterostructures in the next-generation optoelectronic devices, flexible electronics, and optoelectronics [26,27].

## 2. The Controlled Growth Preparation Experiment and Transfer of h-BN

The platinum (Pt) sheet with a thickness of 20 µm and a purity of 99.95% is selected in the growth experiment of h-BN. This is because Pt substrate has the smooth atomic surface, which is conducive to the growth of high-quality, uniform h-BN thin film [28]. Besides, Pt substrate can also be reused by using the bubble transfer method. The following describes cleaning processes of Pt substrate. First, Pt sheet is cut to a size of 1.5 × l cm. Then, these Pt sheets are put successively in the acetone and alcohol solution for the ultrasonic cleaning of 10 min, which can remove organic matter and impurities on the surface of Pt sheets. Finally, Pt sheets are blown dry with nitrogen gas.

**Figure 1:**
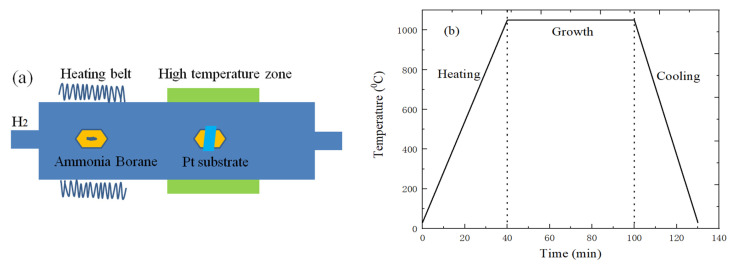
(**a**) Growth schematic diagram; (**b**) CVD temperature change curve of h-BN preparation.

Figure 1a is the growth schematic diagram of h-BN on Pt substrate by CVD method. The ammonia borane precursor powder is heated by heating belt, and it can generate Hydrogen gas (H_2_), Aminoborane Polymer and Borazine [29]. As a solid material, aminoborane polymer remains in the quartz boat, and Borazane enters the high temperature heating zone of furnace under H_2_ gas. Meanwhile, Borazane can be dehydrogenated again under the catalytic action of Pt, so the B and N atoms combine to form h-BN. The preparation of h-BN would be affected by the precursor ammonia borane powder amount, heated temperature and growth time [30]. The h-BN film with different sizes, morphologies and nucleation density can be prepared by changing growth conditions.

The ammonia borane powder is, respectively, placed and wrapped in a U-shaped quartz boat and copper foil, and it is placed on the intake side in the air flow direction, which can decrease the amount of aminoborane polymer particles deposited on the surface of Pt substrate. At the same time, the open quartz boat with cleaned Pt substrate is placed in the high temperature heating zone of the CVD system. The following describe the specific growth processes of h-BN. The heating zone of the CVD tube furnace is first heated to 1050 °C, and Pt substrate is annealed and recrystallized for 30 min. Next, the ammonia borane powder can be heated by the heating belt. When entering the growth stage of h-BN, it is necessary to maintain the appropriate amount of H_2_; the specific growth temperature changes of h-BN are shown in Figure 1b. Finally, the heating belt and furnace power supply need to be quickly cut off when the growth of h-BN is over, and enter the cooling stage.

The following are the specific transfer processes of h-BN material [31]. First, PMMA polymer with 3% mass score is suspended successively on the surface of h-BN/Pt substrate in the rotating speed with 500 rpm of 10 s and 1500 rpm of 20 s, and the h-BN with PMMA polymer is put on the heating table at 100 °C for 5 min, which can cure the PMMA polymer. Then, the PMMA/h-BN/Pt sample is immersed in the aqua regia solution (1 mol/L) for 1 h, and PMMA/h-BN would float on the surface of solution when Pt substrate is etched. Next, PMMA/h-BN sample is fished in deionized water and allowed to stand for 30 min, and the operation is repeated three times to clean h-BN. Subsequently, PMMA/h-BN is picked up with SiO_2_/Si substrate, and the sample is placed on hot plate. It is heated at 60 °C to remove the water between PMMA/h-BN and SiO_2_/Si substrate, which would promote h-BN material and SiO_2_/Si substrate combined closely. Afterwards, PMMA/h-BN/SiO_2_/Si sample is immersed in acetone solution for 30 min, then the acetone solution is replaced and allowed to stand for 12 h, before the h-BN/SiO_2_/Si sample is blown dry with nitrogen gas. Finally, h-BN/SiO_2_/Si sample is annealed at 400 °C for 1 h, which removes the PMMA polymer, water and other impurities on the surface of h-BN.

## 3. The Preparation and Characterization of TMDs/h-BN Heterostructure

**Figure 2:**
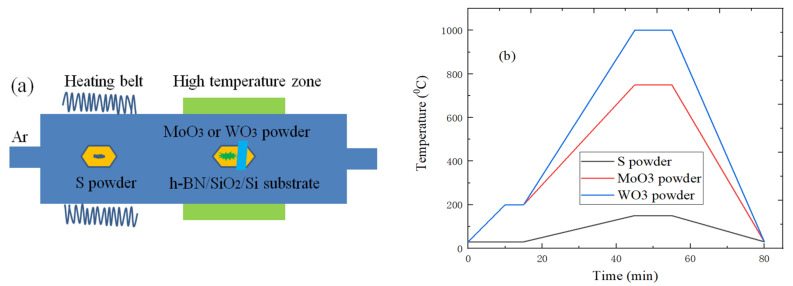
(**a**) CVD growth experiment schematic diagram; (**b**) Temperature change curve of TMDs/h-BN heterostructure.

Figure 2a shows the preparation schematic diagram of TMDs/h-BN heterostructure, which can be achieved by using APCVD method to grow MoS_2_ and WS_2_ on h-BN/SiO_2_/Si substrate. The S powder, WO_3_ or MoO_3_ powder are, respectively, put into two quartz boats, h-BN/SiO_2_/Si substrate is placed on the top of quartz boat with WO_3_ (or MoO_3_) powder, and the above quartz boats are sent to the corresponding position of tube furnace, wherein the quartz boats with 100 mg S powder and 2 mg WO_3_ (or MoO_3_) powder are, respectively, placed in the area 1 and area 2, as show in Figure 2a. Additionally, the high-purity argon gas is used as carrier gas during the growth process. In Figure 2b, each temperature zone of the CVD system is set to the corresponding reactants temperature, the temperature of S powder is set to 150 °C, and the WO_3_ or MoO_3_ powders are 1000 °C and 750 °C, respectively. It is necessary to continuously provide 300 sccm Ar gas for 20 min before heating to exhaust the air and purify CVD growth system. Subsequently, the flow rate of Ar gas is adjusted to 50 sccm, and the growth time is maintained for 10 min. The S powder would evaporate and react with the MoO_3_ or WO_3_ powders during the growth processes. The competitive processes of sublimation, reaction, transfer, diffusion and precipitation can be balanced, which is beneficial to the dense growth of WS_2_ and MoS_2_ materials. When the chemical reaction is over, the system can naturally cool to room temperature. At the same time, it is necessary to continue to provide Ar gas to eliminate the residual gas of tube furnace. The temperature of S powder, WO_3_ or MoO_3_ powders can be separately controlled by tube furnace, and the supply rate of reactants can also be controlled by furnace temperature. In order to stably provide the optimal rate for each reactant material, it is crucial to independently control the supply rate and growth temperature. The TMDs material growth is completed under the optimal conditions, which would improve the operability of the reaction process.

The morphology and size of WS_2_/h-BN and MoS_2_/h-BN heterostructure materials (SixCarbon Technology Shenzhen, Shenzhen, China) can be observed and measured by the optical microscope, Atomic force microscopy (AFM), Raman and photoluminescence (PL) spectroscopy, which can study the structure, film thickness, internal and external strains of TMDs/h-BN heterostructures [32]. The Raman and PL spectroscopy analysis are carried out with the high-resolution dispersion Raman spectrometer, and the corresponding test condition is under the 532 nm laser with a spatial resolution of 1 µm. The horizontal and vertical spatial resolution is 1 and 2 µm, respectively. The 100× objective lens is used to focus the laser beam onto the TMDs/h-BN heterostructure in this experiment, and scattered light can also be collected by the objective lens. The excitation power is less than 1 mW, and the laser power is adjusted from 0.1% to 100% continuously and automatically, which can achieve the accurate measurement of Raman and PL spectrum. The notch filter is used to filter out the Rayleigh radiation, and the charge-coupled device (CCD) is also used to detect Raman and PL signals. The focal length, scanning speed, Raman filter, and the lowest wave number are 800 mm, 3 µs/pixel, 50 cm^−1^ and 10 cm^−1^. The Raman measurement range is 0–1500 cm^−1^, and the photoluminescence spectrum measurement range is 550–800 nm. All optical characterizations are carried out under the normal pressure and temperature.

## 4. The Characterization of MoS_2_/h-BN Heterostructure

### 4.1. The Optical Micrograph of MoS_2_/h-BN Heterostructure

**Figure 3:**
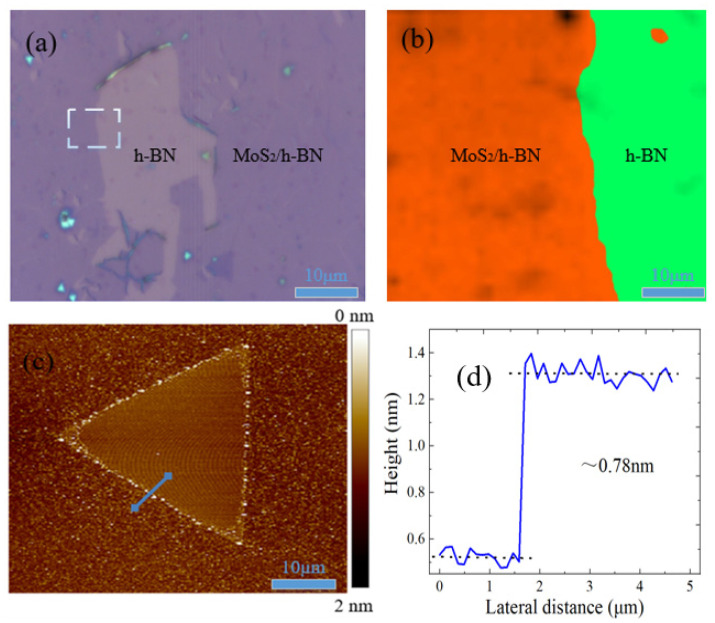
(**a**) Optical micrograph, (**b**) Raman spectrum mapping, (**c**) AFM and (**d**) height profile of MoS_2_/h-BN heterostructure on SiO_2_/Si substrate.

Although the lattice constants of two materials are highly mismatched, the vdW epitaxy technology can still cause one type of 2D material to grow on another material through the rotationally proportional manner, which can form the TMDs/h-BN heterostructure materials with oriented lattice match. Figure 3 shows the optical microscope images of MoS_2_/h-BN heterostructure on SiO_2_/Si substrate at different position. The growth mode of MoS_2_ on h-BN is Frank Van der Merwe mechanism, and MoS_2_ would first form a small 2D nucleus and then grow into the large 2D crystal. In addition, the clean and smooth surface of h-BN is suitable to the CVD growth of single crystal MoS_2_, and it can determine the crystal orientation, which is conducive to form the continuous film. Additionally, the low relative rotation angle between MoS_2_ and h-BN can be attributed to vdW epitaxy, which can be affected by the Coulomb interaction and vdWs force. Figure 3b is the Raman spectrum mapping of MoS_2_/h-BN heterojunction; there are few defects, the fluorescence efficiency is very high, and the quality and uniformity of heterojunction sample are very uniform and good, respectively. Besides, AFM is the most commonly used test method to characterize the thickness of nanomaterials. It can be found by observing Figure 3c,d that the height difference between the sample surface of MoS_2_ material and the surface of h-BN/SiO_2_/Si substrate is 0.78 nm, which can be judged as the monolayer MoS_2_ material.

### 4.2. The Spectral Characteristics of h-BN on SiO_2_/Si Substrate

**Figure 4:**
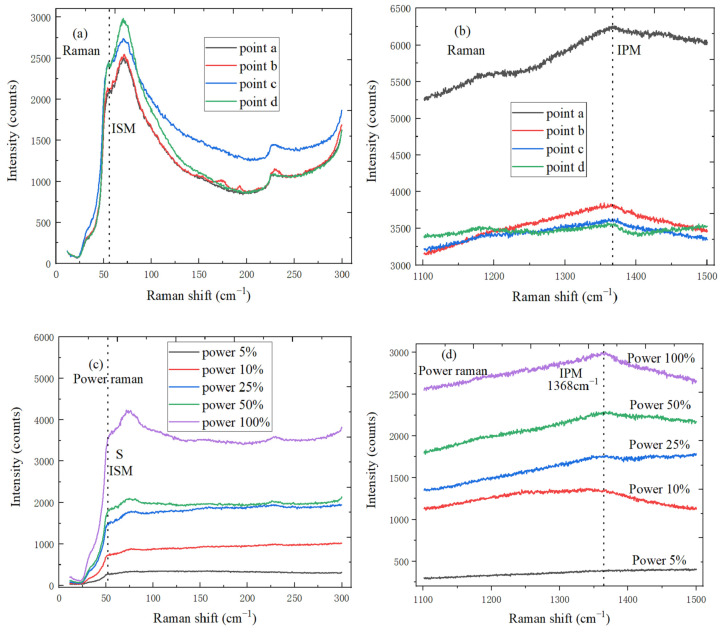
(**a**) ISM mode characteristic peaks of h-BN at different test points; (**b**) IPM mode characteristic peaks of h-BN at different test points; (**c**) ISM mode characteristic peaks of h-BN under the different laser power; (**d**) IPM mode characteristic peaks of h-BN under the different laser power.

Raman spectroscopy is used to characterize and analyze the transferred h-BN material; points a, b, c, and d in Figure 4a,b are selected from the different h-BN material regions on SiO_2_/Si substrate, the ultra-low frequency line at 54.5 cm^−1^ and the high-frequency line at 1361 cm^−1^, respectively, correspond to the interlayer shear mode (ISM) with E_2g_ symmetry and in-plane mode (IPM). Since both modes belong to the same irreducible representation, the huge difference of intensity is interpreted as Raman tensor, and crystallinity of the transferred h-BN is very high, which is the energy difference sign between weak interlayer interaction and atomic interaction of the strong plane [33]. The peak position of ISM mode change with the laser power increases, as show in Figure 4c. In the thinner h-BN sheet, the thermal effect is more obvious. When the higher laser power is used on the h-BN sample, the temperature would rise, and the peak position would cause the additional frequency shift. In addition, Raman process of h-BN is non-resonant when the laser source is in visible range. The ISM Raman signal of nanoscale layer hBN is much weaker than that of other 2D materials, so the longer integration time need be required to minimize the noise level. In Figure 4d, the temperature around h-BN sample increases with laser power increases, IPM frequency changes linearly with temperature, and the IPM mode peak intensity also increases. The reason is that phonon–phonon interaction can generate the non-harmonicity, peak position of IPM mode is more temperature dependent, and IPM mode frequency is more sensitive to the sample heating.

### 4.3. The Spectral Characteristics of h-BN with Different Layers on SiO_2_/Si Substrate

**Figure 5:**
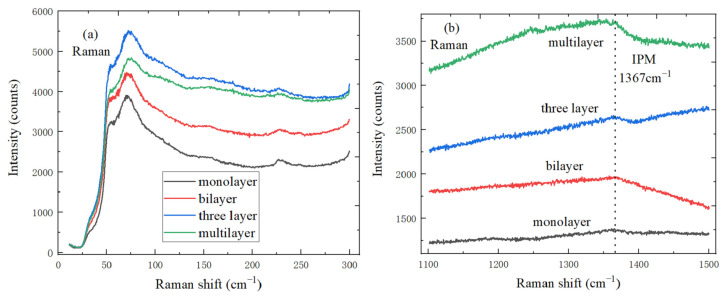
Spectral characteristics of h-BN with different layers (**a**) ISM mode characteristic peak spectrum; (**b**) IPM mode characteristic peak spectrum.

Figure 5a plots the group Raman spectrum of h-BN samples from monolayer to multi-layer, the Stokes spectrum is also plotted in low frequency region, and peak position of ISM shear mode would strongly move down when the thickness decrease. In Figure 5b, the peak position of IPM mode characteristic peak is at 1367 cm^−1^, IPM frequency shift does not change significantly with the layer number, and Raman signal is stronger when h-BN film is thicker [34].

### 4.4. The Spectral Characteristics of MoS_2_ on SiO_2_/Si Substrate

The Raman and PL spectroscopy are used to analyze the physical properties of MoS_2_, and PL emission spectroscopy is a powerful tool for studying the energy band structure and electronic excitation. The neutral excitons can be generated by the Coulomb interaction between an electron and a hole, and the excitons are charged by combining another electron or hole. The Raman and PL spectrum phenomena are largely dependent on temperature, which can lead to the thermal expansion/contraction of lattice and anharmonic interaction between phonon modes.

**Figure 6:**
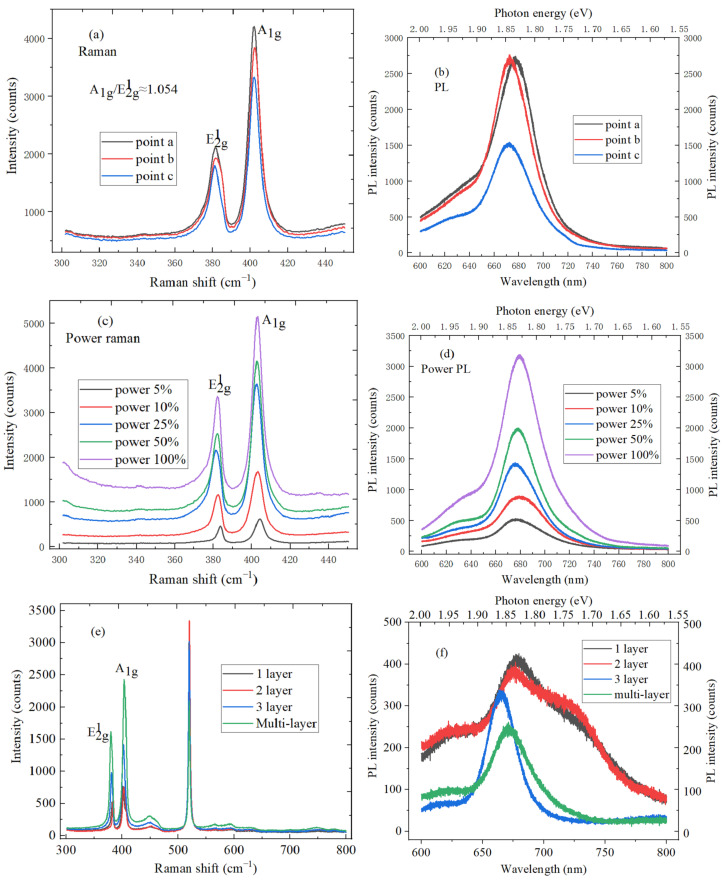
(**a**) Raman spectrum of MoS_2_ at three different test points; (**b**) PL spectrum of MoS_2_ at three different test points; (**c**) Raman spectrum of MoS_2_ under the different laser power; (**d**) PL spectrum of MoS_2_ under the different laser power; (**e**) Raman spectrum of MoS_2_ material with different layers; (**f**) Raman spectrum of MoS_2_ material with different layers.

The Raman peak of silicon is located at 520 cm^−1^ under 532 nm excitation wavelength at the room temperature, and Raman peak frequency of silicon represents the actual temperature, which can calibrate the spectrums. The Raman and PL spectroscopy tests were performed at the three different test points of MoS_2_ material on SiO_2_/Si substrate. In Figure 6a, E^1^_2g_ and A_1g_ mode characteristic peaks can be observed under the non-resonant condition of 532 nm laser, the in-plane E^1^_2g_ mode comes from the opposite vibration of Mo atoms relative to two S atoms, while A_lg_ mode participates in the out-of-plane vibration of S atoms in opposite directions. The layer dependence between E^1^_2g_ and A_1g_ mode characteristic peaks is mainly due to the long-term Coulomb interaction and the interlayer vdW force. The E^1^_2g_ and A_1g_ mode characteristic peaks are, respectively, located at 381.7 and 400.3 cm^−1^, the distance is 18.6 cm^−1^, and the ratio of A_1g_/E^1^_2g_ is about 1.05, which indicates the existence of monolayer MoS_2_. It can be found by observing Figure 6b that PL spectrum is fitted by the Lorentz function, the peak position of the strongest PL intensity is located at 672.2 nm, and the corresponding band gap width is 1.85 eV, which is consistent with the direct band gap of monolayer MoS_2_. Monolayer MoS_2_ material shows the direct electron band gap of 1.85 eV, multi-layer MoS_2_ is the smaller indirect gap material, and the transition can greatly improve the quantum yield of PL spectrum. Figure 6c is the power Raman spectrum of MoS_2_, the peak intensity of the Raman spectrum gradually increases with the laser power increases, and E^1^_2g_ and A_1g_ mode characteristic peak positions are the blue-shifted. The reason is that the temperature change of MoS_2_ would cause the non-harmonic interaction between phonon modes, thermal expansion and the contraction of lattice when the laser power increases. As the electron-phonon coupling increases, PL peak energy appears the red-shifted with temperature increases, as shown in Figure 6d. Additionally, PL spectrum intensity increases with the laser power increases. The resonance Raman scattering of MoS_2_ is studied by matching the excitation energy to PL spectrum exciton peak energy of MoS_2_, which can help to understand and master the energy band structure and exciton transition. Figure 6e shows the Raman spectrum of MoS_2_ material with different layers, the peak spacing between E^1^_2g_ and A_1g_ characteristic peaks increases with the layer number of MoS_2_ material increases, which can be used to judge the layer number of the MoS_2_ material. It can be found by observing Figure 6f that the characteristic peak intensity decreases with the layers number of the MoS_2_ material increases. The direct gap upper limit of MoS_2_ material is 1.87 eV under our experimental conditions, and the peak position of I characteristic peak has a red shift to a certain extent with the layer number decreases, which can be explained by the vdWs interaction force.

### 4.5. The Spectral Characteristics of MoS_2_/h-BN Heterostructure on SiO_2_/Si Substrate

The Raman and PL spectroscopy are used to explore the lattice strain effects, doping levels and the stacking interactions of heterostructure, which can estimate the quality of MoS_2_/h-BN heterostructure material. Furthermore, measurement results can be analyzed and compared with that of monolayer MoS_2_ grown on SiO_2_/Si substrate by CVD.

**Figure 7:**
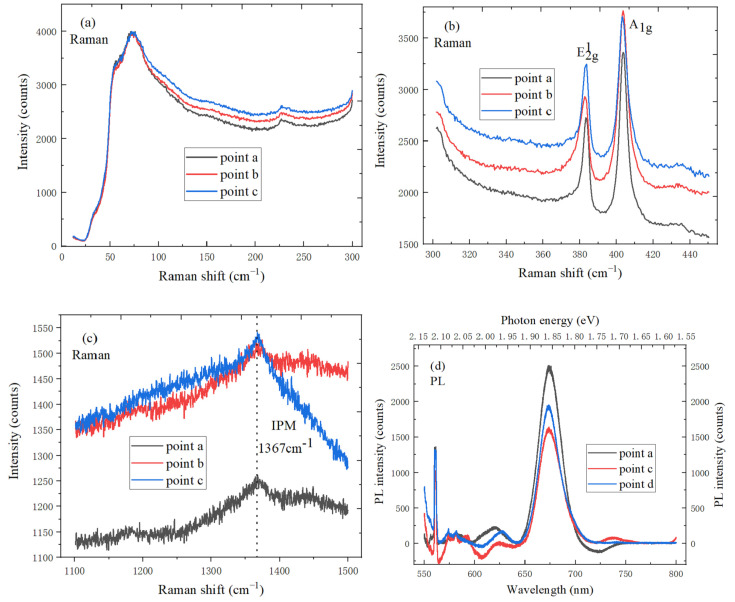

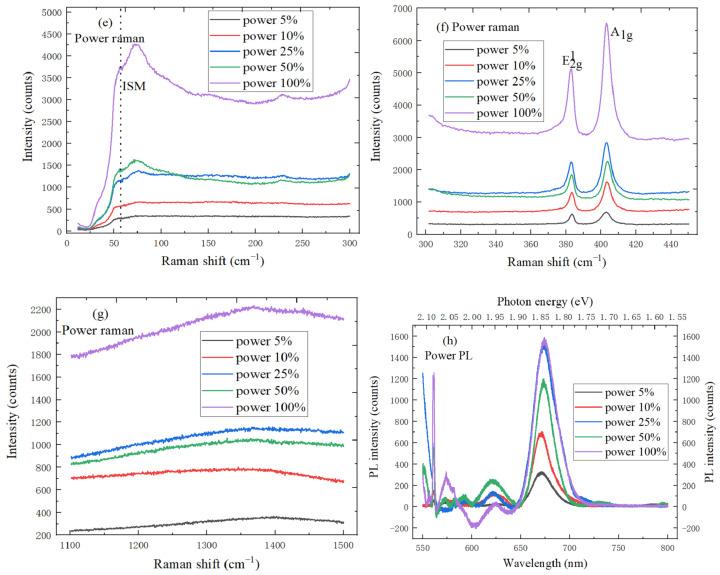
Spectral characteristics of MoS_2_/h-BN heterostructure. (**a**) ISM mode peak of h-BN at three different test points; (**b**) Raman peak of MoS_2_ at three different test points; (**c**) IPM mode peak of h-BN at three different test points; (**d**) PL spectrum of MoS_2_/h-BN heterostructure at three different test points; (**e**) ISM power mode peak of h-BN; (**f**) power Raman peak of MoS_2_; (**g**) IPM power mode peak of h-BN; (**h**) power PL spectrum of MoS_2_/h-BN heterostructure.

Figure 7a shows the ISM mode peak of h-BN at three different test points in the CVD-grown MoS_2_ domain, the ISM mode peak position is at 55.6 cm^‒1^, and it has a red shift, which can confirm the existence of h-BN film after the growth of TMDs material. Figure 7b shows the Raman spectra of MoS_2_ at three different test points, the blue shift of E^1^_2g_ peak position is about 2.8 cm^−1^, and the lattice change can be easily released when the CVD-grown h-BN film is used as substrate. The blue shift of MoS_2_/h-BN heterostructure is about 1.2 cm^‒1^ compared to A_1g_ peak position of MoS_2_/SiO_2_, and the doping level is reduced. The h-BN substrate can introduce the local strain, charged impurities and vdWs interaction, and the electron density between heterostructure interfaces decreases, which has the external effect on Raman spectrum of MoS_2_. In Figure 7c, IPM mode characteristic peak of h-BN at three different test points is located at 1367 cm^‒1^, which can also confirm the existence of h-BN film after the growth of TMDs material. As shown in Figure 7d, PL spectrum of MoS_2_/h-BN heterostructure at three different test points reveal the band structure and exciton characteristics. Due to the substrate effect, PL peak energy of MoS_2_/h-BN is blue-shifted by 10 meV compared to MoS_2_/SiO_2_/Si substrate. The tensile strain and charge doping can induce the red shift of MoS_2_ PL spectrum by changing the energy band structure and electron-phonon coupling. Therefore, PL peak position would be blue-shifted by reducing the local strain and the charge doping of impurities on h-BN substrate. The ISM mode characteristic peak intensity of h-BN increases with the laser power increases, full width at half maximum (FWHM) of characteristic peak decreases, and the peak position shifts red, as shown in Figure 7e. The above results indicate that the directly grown MoS_2_/h-BN heterostructure has the tighter interlayer contact and lower charged impurities.

Figure 7f shows the power Raman spectrum of MoS_2_. The position shift of the characteristic peak varies with temperature, which is due to the temperature-dependent electron-phonon coupling and vdW interaction of MoS_2_/h-BN heterostructure. The E^1^_2g_ mode frequency of MoS_2_ becomes inversely proportional to the applied strain when the laser power increases, and the h-BN substrate appears with the blueshift, which is due to the reduced strain. The A_lg_ mode frequency is also inversely related to the charge doping of MoS_2_, and A_lg_ mode characteristic peak of MoS_2_ on h-BN is blue-shifted by 0.5 cm^−1^ compared with SiO_2_ substrate. In Figure 7g, IPM mode characteristic peak intensity of h-BN increases with the laser power increases, and the peak position shifts blue. Figure 7h shows the power PL spectrum of MoS_2_/h-BN heterostructure on SiO_2_/Si substrate. The characteristic peak intensity increases with the laser power increases, and the peak position shifts red when the temperature increases. Compared to the PL peak of monolayer MoS_2_/SiO_2_/Si substrate, the PL peak band gap of monolayer MoS_2_ grown on h-BN is closer to the mechanical peeling independent MoS_2_ sheet, peak position is blue-shifted, and the PL peak FWHM of MoS_2_/h-BN is smaller than that of MoS_2_/SiO_2_/Si substrate.

## 5. The Characterization of WS_2_/h-BN Heterostructure

### 5.1. The Optical Micrograph of WS_2_/h-BN Heterostructure

There are many dangling bonds at the edge of thin h-BN substrate, which can provide the nucleation sites during the growth of WS_2_. The unique crystallographic relationship indicates that there is the interaction between WS_2_ and h-BN. Due to the different electronegativity between B and N atoms, the charge density is polarized towards N atom. In addition to vdW forces, Coulomb interaction can also determine the orientation of WS_2_ on hBN.

**Figure 8:**
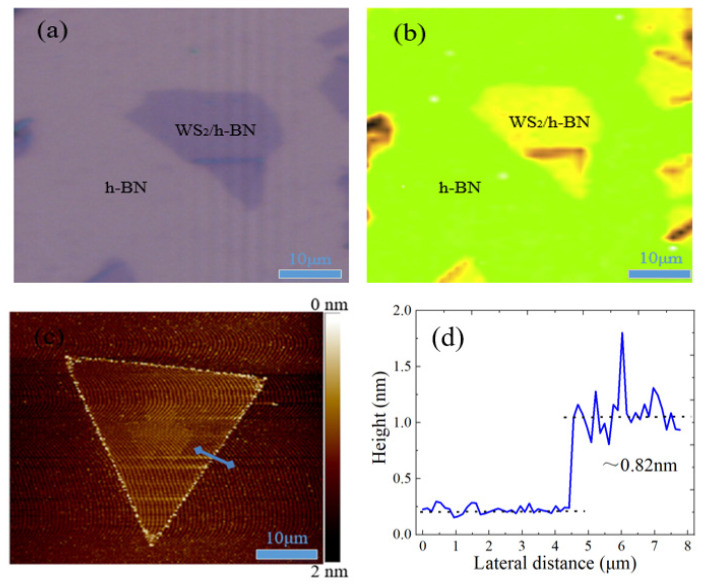
(**a**) Optical micrograph, (**b**) Raman spectrum mapping, (**c**) AFM and (**d**) height profile of WS_2_/h-BN heterostructure on SiO_2_/Si substrate.

Due to the optical contrast, the surface morphology of WS_2_ nanosheets on h-BN/SiO_2_/Si substrate can be clearly observed by the optical microscope and AFM. Figure 8 shows the optical micrograph of WS_2_/h-BN heterostructure at different positions on SiO_2_/Si substrate, most region of the WS_2_ nanosheets have the good morphology and relatively uniform growth, which shows that the WS_2_ material has good film quality. Raman spectroscopy is an effective means to determine the strain distribution state of materials, the Raman spectrum mapping of WS_2_/h-BN heterojunction material was tested at the laser wavelength of 532 nm, and the uniform intensity distribution indicates that the homogeneity of heterojunction sample is very good, as shown in Figure 8b. The surface structure and properties of WS_2_/h-BN heterostructure sample were also characterized by AFM. In Figure 8c,d, the sample surface of WS_2_/h-BN heterojunction film is clean, there are lower roughnesses and defects, and the thickness of WS_2_ material is 0.82 nm, which indicates the presence of monolayer material.

### 5.2. The Spectral Characteristics of WS_2_

**Figure 9:**
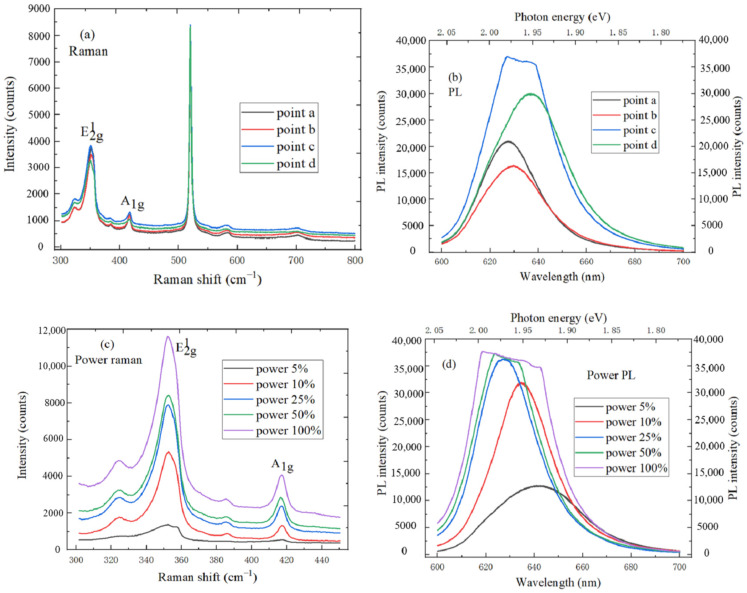
(**a**) Raman spectrum of WS_2_ at four different test points; (**b**) PL spectrum of WS_2_ at four different test points; (**c**) Power Raman spectrum of WS_2_; (**d**) Power PL spectrum of WS_2._

Figure 9a is the Raman spectrum of monolayer WS_2_ on SiO_2_/Si substrate under the four different test points, which is excited by 532 nm laser wavelength. The frequency difference between E^1^_2g_ and A_1g_ modes characteristic peak decreases monotonously as the film thickness decreases, which can identify the layer number of WS_2_. The characteristic peak of E^1^_2g_ in-plane vibration mode and A_1g_ out-of-plane vibration mode are, respectively, located at 353.5 cm^−1^ and 417.6 cm^−1^, the frequency difference is 64.1 cm^−1^, and the existence of monolayer WS_2_ material can be proved by observing Table 1. Figure 9b shows PL spectrum of WS_2_ at four different test points, and the strongest peak position of PL spectrum is 626 nm. It has a long exciton lifetime, coherence time, and the direct optical band gap of 1.98 eV at K and K′ symmetry points in the Brillouin zone, which can cause a significant PL phenomenon. In Figure 9c, the characteristic peak intensity of Raman spectrum accordingly increases as the laser power increases. As shown in Figure 9d, the PL intensity of WS_2_ increases as the laser power increases, and the strongest luminescence peak position shifts blue. The intensity of strongest PL spectrum no longer changes, and the FWHM increases when laser power exceeds 50%. This is because temperature increases as the laser power increases, and the dielectric shielding and exciton excitation effects of WS_2_ material can be weakened.

### 5.3. The Spectral Characteristics of WS_2_/h-BN Heterostructure

**Figure 10:**
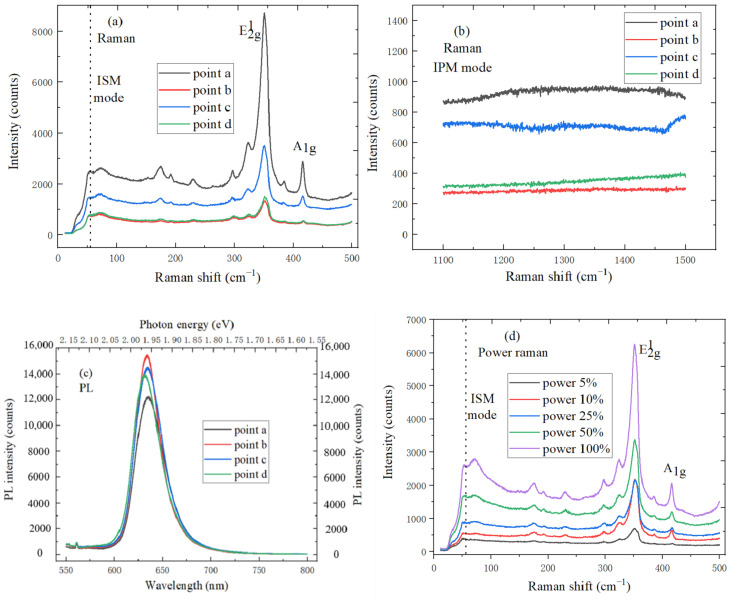

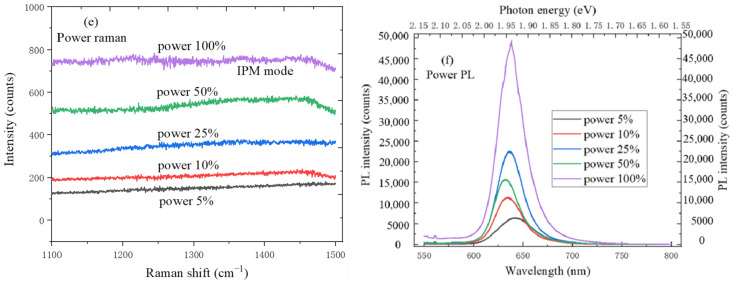
Spectral characteristics of WS_2_/h-BN heterostructure (**a**) Raman characteristic peaks of h-BN ISM mode and MoS_2_ at four different teat points; (**b**) IPM mode characteristic peaks of h-BN at four different teat points; (**c**) PL spectrum of WS_2_/h-BN heterostructure at four different teat points; (**d**) power Raman characteristic peaks of h-BN ISM mode and MoS_2_; (**e**) IPM mode power characteristic peaks of h-BN; (**f**) power PL spectrum of WS_2_/h-BN heterostructure.

Figure 10a,b both show the Raman spectrum of WS_2_/h-BN heterostructure at four different test points, which is measured with an excitation wavelength of 532 nm. The E^1^_2g_ and A_1g_ Raman activation mode characteristic peaks are, respectively, located at 356.3 and 416.6 cm^−1^, and the spacing is 60.3 cm^−1^, which corresponds to the reported peak spacing of monolayer WS_2_. Peak position movement is affected by the interaction between layers, and the E^1^_2g_ and A_1g_ mode characteristic peaks are, respectively, red-shifted and blue-shifted when the layers number decreases. In addition, the IPM mode characteristic peak shape of WS_2_/h-BN is not obvious, the difference is attributed to the Van der Waals interaction between layers, and WS_2_ has the effect on the spectrum of h-BN. Figure 10c shows the PL spectrum of WS_2_/h-BN heterostructure at four different test points, the peak position of strongest PL spectrum is located at 1.96 eV, and the FWHM is only 56 meV, which is smaller than that of WS_2_. The FWHM of PL emission peak is related to the exciton lifetime and interface quality, and the WS_2_/h-BN heterostructure material has the higher crystallinity and cleaner interface. In addition, PL peak of WS_2_/h-BN is much brighter than that of WS_2_, so the hBN substrate plays the important role in the formation of high-quality thin WS_2_/h-BN heterostructure material. Figure 10d,e show the power Raman characteristic peaks of WS_2_/h-BN heterostructure, the intensities of ISM, IPM, E^1^_2g_ and A_1g_ modes characteristic peaks accordingly increase as the laser power increases. The Raman vibration peak shape of E^1^_2g_ and A_1g_ modes is sharp, which indicates that the prepared triangular WS_2_ nanosheets have good crystal quality. The A_lg_ phonon wavenumber increases as the laser power increases, and the E^1^_2g_ mode wavenumber decreases. It can be considered as monolayer WS_2_ when A_lg_ intensity is weak. This is because the coupling between electrons and phonons can strongly affect the A_lg_ phonon of monolayer WS_2_, and the A_lg_ peak intensity can be used to determine the thickness of the layer. Additionally, IPM mode characteristic peak intensity of h-BN also increases accordingly as the laser power increases. Figure 10f shows the strong PL spectrum of monolayer WS_2_, PL spectrum mainly comes from the charged exciton peak, peak position is 633 nm, and the corresponding photon energy is 1.96 eV, which is consistent with the direct band gap of monolayer WS_2_. Furthermore, the maximum FWHM is 74 meV, and the crystal quality of WS_2_ is high. As laser power increases, the peak intensity of strongest PL spectrum increases, and the peak position shifts blue.

## 6. Conclusions

In this paper, the general preparation methods of vdW heterostructure are provided. Monolayer MoS_2_ or WS_2_ can be directly grown on h-BN/SiO_2_/Si substrate by APCVD method, which can prepare TMDs/h-BN heterostructure. The test characterization of MoS_2_/h-BN and WS_2_/h-BN vdW heterostructure materials can be accomplished by the optical microscope, AFM, Raman and PL spectroscopy, and the TMDs/h-BN heterostructure has the tighter interlayer contact, clearer interface, smaller lattice strain and the lower doping level. The Raman characteristic peak signal intensity increases with the thickness of h-BN material increase. Raman and PL spectrum peak positions of MoS_2_/h-BN heterostructure show the blueshift compared with the spectrum of MoS_2_ or WS_2_ on SiO_2_/Si substrate. The reason is that there is the local strain and charged impurities, which can be caused by the h-BN substrate and the vdW heterostructure interaction. MoS_2_/h-BN heterostructure show the strong PL peak at 1.85 eV, which is closer to the mechanical peeling MoS_2_. The electron-phonon coupling and vdW interaction of MoS_2_/h-BN heterostructure can be enhanced by reducing the local strain and charged impurities. Furthermore, the strong and sharp PL emission peak of the WS_2_/h-BN heterostructure material is at 1.96 eV, and the FWHM of emission peak is only 56 meV, which is much smaller than the reported value. The quality of TMDs/hBN heterostructures is very high. In addition, IPM mode characteristic peak shape and peak position of WS_2_/h-BN is not obvious, and the difference is attributed to Van der Waals interaction. This research can prepare a variety of novel 2D heterostructures, and improve the basic interlayer coupling understanding of TMDs/h-BN heterostructure, which can provide guidance for the further application of vdW heterostructures in electronic and optoelectronic devices.

## Figures and Tables

**Table 1:** A_1g_ peak and E^1^_2g_ peak positions of different WS_2_ layers.

	1-Layers	2-Layers	3-Layers	Bulk
E^1^_2g_(Γ) (cm^−^^1^)	352.1	350.9	349.8	348.7
A_1g_(Γ) (cm^−^^1^)	417.5	418.3	418.7	419.1
